# Performance of body mass index in predicting diabetes and hypertension in the Eastern Province of Saudi Arabia

**DOI:** 10.4103/0256-4947.57165

**Published:** 2009

**Authors:** Ali M. Almajwal, Nadira A. Al-Baghli, Marijka J. Batterham, Peter G. Williams, Khalid A. Al-Turki, Aqeel J. Al-Ghamdi

**Affiliations:** a From the School of Health Sciences, University of Wollongong, Wollongong, NSW, Australia; b From the College of Applied Medical Sciences, King Saud University, Saudi Arabia; c From the Directorate of Health Affairs, Ministry of Health, Dammam, Saudi Arabia

## Abstract

**BACKGROUND AND OBJECTIVES:**

Body mass index (BMI) is the most widely used measure to define obesity and predict its complications, such as diabetes and hypertension, but its accuracy and usefulness in Saudi subjects is unknown. This study aimed to assess the validity of standard BMI cut-point values in the Saudi population.

**SUBJECTS AND METHODS:**

197 681 adults participated in a cross-sectional study to detect diabetes and hypertension in the Saudi Eastern province in 2004/2005, with blood pressure, fasting blood sugar, height and weight measurements taken. Sensitivities, specificities, areas under the curves, predictive values, likelihood ratios, false positive, false negatives and total misclassification ratios were calculated for various BMI values determined from receiver operating characteristic (ROC) curves. The significance of the association between risk factors and BMI was assessed using regression analysis.

**RESULTS:**

For the definition of overweight, ROC curve analysis suggested optimal BMI cut-offs of 28.50 to 29.50 in men and 30.50 to 31.50 in women, but the levels of sensitivity and specificity were too low to be of clinical value and the overall misclassification was unacceptably high across all the selected BMI values (>0.80). The relationship between BMI and the presence of diabetes and/or hypertension was not improved when a BMI of 25 was used. Using regression analyses, the odds ratios for hypertension and/or diabetes increased significantly from BMI values as low as 21-23 with no improvement in the diagnostic performance of BMI at these cutoffs.

**CONCLUSION:**

In Saudi population, there is an increased risk of diabetes and hypertension relative to BMI, starting at a BMI as low as 21 but overall there is no cutoff BMI level with high predictive value for the development of these chronic diseases, including the WHO definition of obesity at BMI of 30.

Body mass index (BMI) is widely used as a method to classify underweight, overweight and obesity. Around three quarters of Saudi dietitians use this tool as outcome measure to assess the success in weight loss.^1^ BMI is defined as the weight in kilograms divided by the square of the height in meters (kg/m^2^). In 1997, the World Health Organization (WHO) proposed cut-off points for classifying overweight and obesity.^2^,^3^ Overweight is classified as BMI ≥25.0 and obesity is classified as BMI ≥30.0. These cutoffs have been identified on the basis of the association between BMI and chronic diseases and mortality.^2^,^4^ Since these criteria were derived from European populations their appropriateness for Non-European populations including the Saudi population, is unclear, and the recent WHO monograph on obesity acknowledged the ‘need for different standards that are “culturally specific”.^2^

It has been demonstrated that Asians have a higher percentage of body fat than Caucasians at the same BMI cut-off levels and the health risks associated with obesity occur at a lower BMI cut-off level than Caucasians.^5^–^12^ There have been a few attempts to investigate the applicability of the WHO BMI cut-offs in Asian and Pacific populations.^12^,^18^ In 2000, the Regional Office for the Western Pacific Region of WHO with the International Association for the Study of Obesity (IASO) and the International Obesity Task Force (IOTF) defined overweight in Asians as a BMI >23.0 and obesity as a BMI >25.0.^13^ In 2004, WHO did not propose a clear BMI cut-off for all Asians, but they indicated that the cut-off points for observed risk varies between BMI of 22.0 to 25.0 in different Asian populations and these values varies between BMI 26.0 to 31.0 for the high risk cut-off.

Even though BMI has been used extensively in research and clinical practice, there are only a few studies examining its diagnostic accuracy and no study has examined this in a large, non-Caucasian adult population. Therefore, the present study aimed to assess the ability of BMI to diagnose obesity relative to metabolic risk factors and to determine the optimal BMI cut-off points that could be used to classify obesity in the Saudi population.

## SUBJECTS AND METHODS

This study used data from a large survey conducted in the Eastern Province of Saudi Arabia in 2004 and 2005. The aim of the survey was the early detection of diabetes and hypertension and a detailed description of the study design and data collection procedures has been published elsewhere.^19^ Briefly, all Saudi residents in the Eastern Province aged 30 years and older were invited to participate in the survey. Pregnant women and non-Saudi people were excluded from the survey. For recruitment, a media campaign was organized in each sector using written material and audiovisual media. In addition, posters were put up on billboards along the streets and public places in the Eastern Province. The estimated target population of Saudi residents in the Eastern province aged ≥30 years was 650 000 individuals.^20^ A total of 197 681 Saudis responded to the campaign's invitation (30.4%) and 195 851 of them had assessments of height and weight, and presence of diabetes and/or hypertension and were included in the analysis of the present project. The survey through this convenience sample was conducted through more than 300 examination posts run by trained nurses and technicians distributed in the Eastern Province of Saudi Arabia, including all Primary Health Care Centers (PHCCs), governmental hospitals, and several private health places, and other venues, in addition to mobile teams who visited the target population in places of work that had more than 30 employees.

Weight was measured to the nearest 0.5 kg using standardized beam weight scales (Detecto scale, Cardinal Scale Mfg Co., USA) and recorded to the lowest unit without footwear and with only light clothes on. Height was measured to the nearest centimeter with the subjects barefoot and standing with the feet together, ensuring the nape, back, calves and with the ankles pressed against the measuring tape, which is part of weighing scale. BMI was calculated as weight in kilograms divided by height in meters squared (kg/m^2^), and standard WHO cut-off values of a BMI ≥25.0 as overweight and a BMI ≥30.0 as obesity were used to define the prevalence. Blood pressure was measured two times while the subject was at rest in a sitting position. The average of the two measurements was accepted if the difference between the values was less than 5 mm Hg. Measurement was taken using standardized mercury sphygmomanometers (Diplomat Presameter 660-360 manufactured by Riester GMBH, Germany) with an appropriate cuff inflated to a pressure approximately 30 mm Hg greater than systolic and with the subject's arm at the level of the heart. The screening test for hypertension was considered positive if the systolic and diastolic blood pressure was ≥140 and/or ≥90 mm Hg, respectively.^21^ The diagnosis of hypertension was made if positive screening was confirmed on a subsequent day, or if there was a history of previous diagnosis, irrespective of the blood pressure reading. Participants who did not come for the confirmatory test were diagnosed as having hypertension if screening test of systolic and diastolic blood pressure was ≥180 and /or ≥110 mmHg, respectively. These relatively high values were chosen to avoid over diagnosing hypertension in participants who might be in rush or anxious from the results of the evaluation.

Whole blood glucose concentration was measured for all participants using uniform portable glucometer machines with a Medisafe Reader (Terumo Co., Tokyo, Japan), based on reflectance photometry, where the glucose was catalytically oxidized by the glucose oxidase and peroxides enzymes with a color change reaction. A screening test was considered to be positive for hyperglycemia if Capillary Fasting Blood Glucose (CFBG) was ≥100 mg/dL (≥5.6 mmol/L) after at least 8 hours of fasting or the Capillary Random Blood Glucose (CRBG) was ≥140 mg/dL (≥7.8 mmol/L) taken without consideration of the time of the last meal.^22^ A CFBG of 100-125 mg/dl (5.6-6.9 mmol/l) and a CRBG of 140-199 mg/dl (7.8-11 mmol/l) were considered to be consistent with impaired fasting glucose (IFG) and impaired glucose tolerance (IGT), respectively. Initial screening test was considered to be consistent with the diagnosis of diabetes if the CFBG was ≥126 mg/dl (≥7.0 mmol/l) or the CRBG was ≥200 mg/dl (≥11.0 mmol/l). Diabetes mellitus was diagnosed either by a positive history of diabetes or through the screening test. All subjects who had been screened positive for hyperglycemia without a history of diabetes were asked to come in fasting for ≥8 hours, on the following day, at the central laboratory, for confirmation of the results by venous blood testing through the measurement of fasting plasma glucose (FPG). Confirmatory FPG was considered to be diagnostic for diabetes if it was ≥126 mg/dL (≥7.0 mmol/L). Participants who did not come for the confirmatory test were diagnosed as having diabetes if screening test of CFBG was ≥200 mg/dl (11.0 mmol/l) or CRBG was ≥270 mg/dl (15.0 mmol/l).

Data were analyzed with SPSS software (version 17.0; SPSS Inc., Chicago, IL). All results are presented as mean (SD) or percentage, where applicable. Data analysis was performed in men and women separately. BMI was stratified in unit of 0.5 for both men and women. A BMI <19.9 was considered as the reference. Logistic regression analysis was used to examine the independent relationship between the stratified BMI and the odds ratio of having diabetes, hypertension, both diabetes and hypertension and either diabetes or hypertension. *P* value<.001 was considered to be significant.

The optimal sensitivity and specificity using different BMI cut-off values to predict the presence of diabetes and/or hypertension were examined by receiver operating characteristic curve (ROC) analysis. A greater area under the curve (AUC) indicates better predictive capability. An AUC=0.5 indicates that the test performs no better than chance, and an AUC=1.0 indicates perfect discrimination. An ideal test is one that reaches the upper left corner of the graph (100% true positives and no false positives). To determine the optimal BMI cutoff points, we computed and searched for the shortest distance between any point on the curve and the top left corner on the y-axis. Distance was estimated at each one-half unit of BMI according to the equation:^23^,^24^ $\text{Distance in ROC curve}=\sqrt{{\left(1-\text{sensitivity}\right)}^{2}+{\left(1-\text{specificity}\right)}^{2}}$

Additional criteria were also used to select cut-offs, including the greater sum of sensitivity and specificity, the smallest misclassification rate, and the significant associations between BMI and risk factors based on the logistic regression. Diagnostic performance of BMI in predicting diabetes and hypertension was assessed by calculating AUC, sensitivity, specificity, predictive values (Positive Predictive Values and Negative Predictive Values), likelihood ratios (LR+ and LR−), false positive (FP), false negative (FN) and the total misclassification rate.

## RESULTS

A total of 195 851 participants (99 946 men and 95 905 women) were included in the analysis. Table 1 shows study population characteristics. The overall mean (SD) BMI of participants was 29.69 (6.00). The mean weight and height for men were 80.45 (15.94) kg and 1.67 (0.07) m and for women were 73.29 (16.1) kg and 1.54 (0.07) m, respectively. The overall prevalence of obesity (BMI≥30), overweight (BMI 25-29.9), diabetes and hypertension were 43.8, 35.1, 17.2 and 15.6%, respectively. Results of the initial screening test for participants with no previous diagnosis of diabetes or hypertension showed that 10.9% of participants had IFG, IGT or diabetes and 9% had hypertension. Analysis also showed that 59.3% of participants with diabetes and 46% of participants with hypertension had another confirmatory test. This means 4.4% and 4.9% of the total sample did not have the confirmatory test for the diagnosis of diabetes and hypertension. However, >70% of participants who did not come for the confirmatory test for diabetes had IFG or IGT. For hypertension, 53.0 % of participants had diastolic blood pressure ranged from 140 to 150 mmHg and 77.3% of participants had diastolic blood pressure ranged from 90 to 100 mmHg.

**Table 1:** Population characteristics (n=195 851).

	Men	Women	Both genders
Weight (kg)	80.45 (15.94)	73.29 (16.1)	76.95 (16.43)
Height (m)	1.67 (0.07)	1.54 (0.07)	1.61 (0.10)
BMI (kg/m^2^)	28.67 (5.26)	30.75 (6.51)	29.69 (6.00)
Obese (%)	36.1	51.8	43.8
Overweight (%)	40.3	29.7	35.1
Diabetes (%)	15.9	18.6	17.2
Hypertension (%)	13.1	18.1	15.6

**Figure 1:**
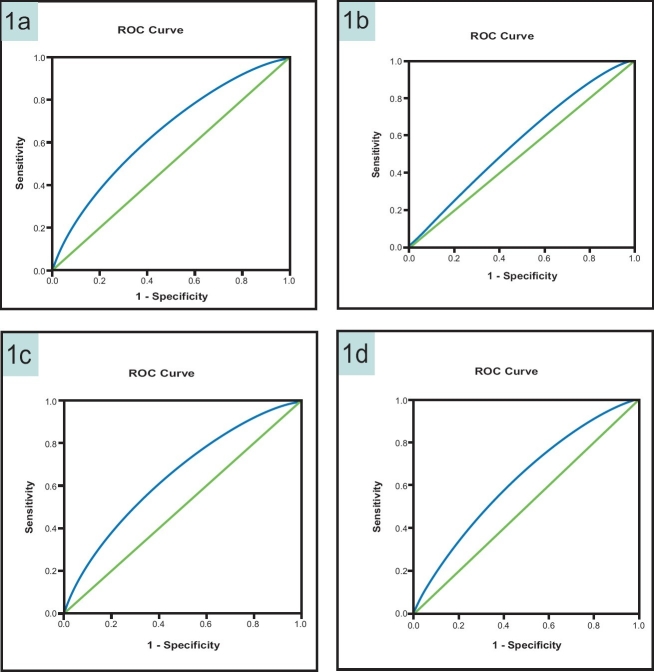
ROC curve showing the performance of BMI in predicting diabetes and hypertension (1a: diabetes in women, AUC=0.618 (95% CI 0.614 to 0.622); 1b: diabetes in men, AUC=0.566 (95% CI 0.561 to 0.571); 1c: hypertension in women, AUC=0.645 (95% CI 0.641 to 0.650); 1d: hypertension in men, AUC=0.625 (95% CI 0.620 to 0.630).

Table 2 displays details of the diagnostic performance of BMI in detecting diabetes and/or hypertension using optimal BMI cut-off values based on the shortest distance in ROC curve. Values ranged from 28.50 to 29.50 in men and from 30.50 to 31.50 in women. The AUC ranged from 0.566 to 0.625 in men and from 0.618 to 0.645 in women (Figure 1). These values were statistically significantly higher than that would be expected by chance alone (*P*<.001).

**Table 2:** Diagnostic performance of BMI in detecting diabetes and/or hypertension using optimal BMI cut-off values based on the shortest distance in ROC curves in Saudi adults, Eastern province, 2004, (n=195 851).

Risk factors	Gender	n	AUC (95% CI)	Cut-offs kg/m^2^	Sensitivity	Specificity	PPV	NPV	LR +	LR −	FP rate	FN rate	Misclassification rate
Diabetes	Men	99946	0.566 (0.561-0.571)	28.50	0.55	0.54	0.19	0.87	1.21	0.82	0.46	0.45	0.91
	Women	95905	0.618 (0.614-0.622)	31.50	0.58	0.61	0.25	0.86	1.48	0.69	0.39	0.42	0.81

Hypertension	Men	99946	0.6 25 (0.62-0.63)	29.00	0.59	0.58	0.18	0.91	1.42	0.70	0.42	0.41	0.83
	Women	95905	0.645 (0.641-0.650)	31.50	0.60	0.62	0.25	0.87	1.55	0.66	0.39	0.41	0.80

Diabetes or Hypertension	Men	99946	0.594 (0.590-0.598)	28.50	0.58	0.56	0.13	0.92	1.30	0.76	0.44	0.42	0.86
	Women	95905	0.640 (0.636-0.643)	30.50	0.63	0.58	0.17	0.92	1.47	0.65	0.43	0.37	0.80

Diabetes and Hypertension	Men	99946	0.618 (0.611-0.625)	29.50	0.55	0.62	0.08	0.96	1.44	0.73	0.38	0.45	0.83
	Women	95905	0.643 (0.637-0.649)	31.50	0.61	0.59	0.12	0.94	1.55	0.66	0.41	0.39	0.80

AUC: area under the curve; PPV: positive predictive value; NPV: negative predictive value; LR+: positive likelihood ratio; LR−: negative likelihood ratio; FP rate: false positive rate; FN rate: false negative rate

The corresponding sensitivities and specificities in men ranged from 0.55 to 0.59 and 0.54 to 0.62, respectively, and in women they ranged from 0.58 to 0.63 and 0.58 to 0.62, respectively. LRs were close to 1.0 in both men and women. The positive likelihood ratio (LR+) ranged from 1.21 to 1.55 and the negative likelihood ratio (LR−) ranged from 0.66 to 0.82. The PPV were small, ranging from 0.08 to 0.25; NPV were high, ranging from 0.87 to 0.96. FP and FN rates were close to each other and ranged from 0.38 to 0.46 and from 0.37 to 0.45, respectively. The overall misclassification was unacceptably high across all the selected BMI values (>.80). These cut-offs were selected based on the shortest distance in the ROC curves. However, when other criteria applied, including the greater sum of sensitivity and specificity, and the smallest misclassification rate, the results were very similar (data not shown).

Table 3 shows the odds ratios of the association between diabetes and hypertension and BMI in men and women. A significant positive association was observed with BMI values starting at 21 to 23 and increasing progressively with higher BMI values for both genders. Table 4 displays the predictive value of BMI in detecting diabetes and/or hypertension using BMI cut-off values based on the lowest significant association between BMI and the risk factors from the logistic regression analysis. The diagnostic performance of BMI was also assessed using a BMI of 25 (the value recommended by WHO to identify overweight), but the results showed poor performance (data not shown).

**Table 3:** Risk of diabetes and/or hypertension associated with increasing BMI in Saudi adults, Eastern province, 2004, based on regression analysis (n=195 851).

BMI	Diabetes odds ratio (95% CI)		Hypertension odds ratio (95% CI)		Diabetes and hypertension odds ratio (95% CI)		Diabetes or hypertension odds ratio (95% CI)	
	Men	Women	Men	Women	Men	Women	Men	Women
21	1.43* (1.16-1.78)	1.28 (0.98-1.67)	1.15 (0.89-1.48)	1.05 (0.82-1.34)	1.47 (0.93-2.30)	1.64 (1.09-2.45)	1.30* (1.08-1.55)	1.05 (0.86-1.29)
22	1.67* (1.38-2.03)	1.63* (1.29-2.05)	1.11 (0.88-1.41)	1.22 (0.98-1.50)	1.59 (1.06-2.40)	1.59 (1.10-2.32)	1.41* (1.20-1.66)	1.37* (1.15-1.63)
23	1.99* (1.67-2.38)	2.00* 1.63-2.47)	1.53* (1.25-1.87)	1.34* (1.10-1.62)	2.21* (1.53-3.17)	2.23* (1.59-3.11)	1.76* (1.53-2.04)	1.53* (1.30-1.79)
24	2.22* (1.88-2.63)	1.99* (1.63-2.44)	1.69* (1.40-2.05)	1.55* (1.29-1.86)	2.47* (1.74-3.49)	2.14* (1.55-2.97)	1.98* (1.72-2.27)	1.70* (1.46-1.97)
25	2.43* (2.07-2.86)	2.71* (2.24-3.28)	2.00* (1.66-2.38)	1.80* (1.51-2.13)	3.02* (2.16-4.21)	2.86* (2.10-3.89)	2.20* (1.93-2.51)	2.12* (1.84-2.45)
26	2.80* (2.39-3.28)	2.85* (2.34-3.44)	2.08* (1.74-2.49)	1.84* (1.55-2.19)	3.26* (2.35-4.53)	2.79* (2.06-3.79)	2.47* (2.17-2.81)	2.25* (1.95-2.59)
27	2.76* (2.36-3.23)	2.85* (2.36-3.44)	2.45* (2.06-2.92)	2.03* (1.72-2.40)	3.69* (2.67-5.12)	3.56* (2.64-4.79)	2.59* (2.28-2.95)	2.51* (2.19-2.88)
28	3.01* (2.57-3.51)	3.37* (2.80-4.05)	2.72* (2.29-3.24)	2.27* (1.93-2.67)	4.54* (3.29-6.26)	4.27* (3.19-5.71)	2.78* (2.45-3.16)	2.85* (2.49-3.27)
29	3.25* (2.78-3.80)	3.94* (3.29-4.73)	2.90* (2.43-3.45)	2.54* (2.16-2.99)	4.31* (3.11-5.95)	4.32* (3.23-5.79)	3.11* (2.74-3.54)	3.39* (2.96-3.88)
30	3.35* (2.87-3.92)	4.50* (3.76-5.39)	3.07* (2.58-3.65)	2.80* (2.39-3.29)	4.77* (3.46-6.59)	4.66* (3.48-6.22)	3.20* (2.82-3.64)	3.68* (3.22-4.20)
31	3.46* (2.96-4.05)	4.73* (3.96-5.66)	3.60* (3.02-4.28)	3.00* (2.56-3.52)	5.89* (4.28-8.12)	5.23* (3.92-6.99)	3.40* (3.00-3.87)	3.79* (3.324.34)

* Odds of disease significant (*P*<.001) when compared with BMI <20 as a reference group

**Table 4:** Diagnostic performance of BMI in detecting diabetes and/or hypertension using optimal BMI cut-off values based on the significant association using logistic regression in Saudi adults, Eastern province, 2004, (n=195 851).

Risk factors	Gender	n	AUC (95% CI)	Cut-offs kg/m^2^	Sensitivity	Specificity	PPV	NPV	LR +	LR −	FP rate	FN rate	Misclassification rate
Diabetes	Men	99946	0.566 (0.561-0.571)	21	0.98	0.06	0.16	0.94	1.04	0.33	0.94	0.02	0.96
	Women	95905	0.618 (0.614-0.622)	22	0.98	0.08	0.20	0.95	1.07	0.25	0.92	0.02	0.94

Hypertension	Men	99946	0.625 (0.62-0.63)	23	0.95	0.13	0.14	0.95	1.09	0.38	0.87	0.05	0.92
	Women	95905	0.645 (0.641-0.650)	23	0.96	0.12	0.19	0.93	1.09	0.33	0.88	0.04	0.92

Diabetes or Hypertension	Men	99946	0.594 (0.590-0.598)	21	0.98	0.06	0.11	0.96	1.04	0.33	0.94	0.02	0.96
	Women	95905	0.640 (0.636-0.643)	22	0.97	0.09	0.13	0.95	1.07	0.33	0.91	0.03	0.94

Diabetes and Hypertension	Men	99946	0.618 (0.611-0.625)	23	0.96	0.12	0.06	0.98	1.09	0.33	0.88	0.04	0.92
	Women	95905	0.643 (0.637-0.649)	23	0.97	0.11	0.09	0.98	1.09	0.33	0.89	0.03	0.92

AUC: area under the curve; PPV: positive predictive value; NPV: negative predictive value; LR+: positive likelihood ratio; LR−: negative likelihood ratio; FP rate: false positive rate; FN rate: false negative rate.

## DISCUSSION

This is the first population-based study to assess the ability of BMI to diagnose obesity and to determine the optimal BMI cut-off points for the Saudi population based on the prevalence of diabetes and hypertension. BMI has been shown to be associated with cardiovascular diseases such as diabetes and hypertension in Caucasians.^25^–^27^ These relationships, which have been used in many studies to assess the accuracy of BMI in diagnosing obesity, have also been demonstrated in Middle Eastern people, including Saudis.^28^–^30^ The use of a reliable tool with optimal cut-off points for obesity diagnosis is very important to establish consequent public health policies, treatment protocols and to determine the correct prevalence of obesity for each population.

ROC curve analysis, using the endpoints of presence of diabetes or hypertension, showed that the optimal BMI cut-off points for overweight ranged from 28.50 to 29.50 for men and from 30.50 to 31.50 for women depending on the risk factor being studied. These values are higher than the suggested values by WHO, particularly in women. One possible reason for the high value for women is the short stature in this group with a mean height of 1.54 m. Lara-Esqueda et al^31^ conducted a large cross-sectional study (n=119, 975) to assess the ability of the BMI to predict obesity-associated morbidity in Mexican participants with normal or short stature. The results showed that the BMI value with the best diagnostic proficiency ranged from 27 to 29 in normal stature women and from 28 to 29 in short stature women. The authors concluded that the proficiency of BMI as a diagnostic test is poor in short stature participants. However, lowering the BMI threshold did not improve the ability of BMI to predict diabetes and hypertension in that study and neither did it in our study as well.

The overall performance of the ROC curve can be quantified by estimating the AUC which ranged from 0.57 to 0.65 (Table 4). An area of 1.0 is perfect and an area <0.5 is considered non-informative. Our results indicated that the ROC analysis was close to a non-informative test (Figure 1). To avoid a misleading conclusion, several other diagnostic characteristics of BMI as a tool for obesity diagnosis were calculated.

ROC curve analysis showed that the corresponding sensitivities and specificities were poor (<0.63 and <0.62, respectively). This indicates that the percentage of people identified as having the risk factors and the percentage of people who were identified as not being at risk were less than 63% of total population. Both LR+ and LR− were close to 1.0, indicating a minimal increase in the likelihood of the presence of the risk factor if the test is positive and a minimal decrease in the likelihood if the test is negative. PPVs were small and ranged from 0.08-0.25. This indicates that the proportion of overweight and obese people who were classified correctly as overweight or obese was <25%. On other hand, the proportion of non-overweight or non-obese people who were classified correctly were ranged from 86% to 96% as indicated by the NPVs. The FP and FN rates were high and close to each other in both women and men. The overall misclassification was very high and exceeded 80% of the total population across all the selected BMI cut-off points.

The technical statistical term “diagnostic performance” has been used to characterize the relationship between BMI and presence of diabetes or hypertension. This does not mean that BMI is used a diagnostic clinical test for these conditions. However, clinicians will generally be more concerned about false negatives, when patients at risk may be overlooked for treatment. By using BMI as a tool for assessing the metabolic risks of being overweight or obese, these findings suggest that 37% to 45% of people with risk factors would be incorrectly identified as healthy, as indicated by the FN rate. The percentage varies depending on the risk factors being studied. This finding is also supported by the high values of specificities and NPVs and the values of LR-. The use of the higher BMI cut-off values suggested by the ROC analysis (Table 2) would misclassify large percentages of people with risk factors as being healthy who might then miss the opportunity for treatment.

To reduce the large chance of such misclassification, we attempted to identify cut-offs based on the observed significant association between BMI and risk factors. Regression analysis showed that the risk of diabetes and/or hypertension was significantly increased at BMI values as low as 21-23 and increased progressively as BMI increased. Applying this criterion to identify the cut-off values resulted in improvements in sensitivity, NPV, LR−, FN and worsening in specificity, PPV, LR+, FP and the overall misclassification rate. Using these lower BMI cut-offs resulted in a very small FN rate ranging from 0.02 to 0.05. Therefore, most people with risk factors were correctly identified as at risk. This finding may suggest obesity management should be considered even at a quite low BMI values in the Saudi population.

This is not the first study to suggest the presence of a significantly increased risk of co-morbidities at BMI values less than 25. In a Chinese population in Hong Kong, diabetes and hypertension were also reported to increase from a BMI value of 22 onwards.^38^ However, the use of such low cut-offs would lead to large misclassification of healthy people as being at risk, as indicted by the high values of sensitivities and FP rates. This fact that could cause unnecessary and costly diagnostic testing. Overall the total misclassification rate was unacceptably high, even with the use of different BMI cut off points and different selection criteria, and even with the use of the recommended value by WHO (BMI=25). These findings illustrate the significant limitations in using BMI alone for obesity diagnosis in the Saudi Arabian population.

To our knowledge, only one study has assessed the optimal BMI cut-off points on a sample of the Arab population of the Middle East, which was conducted in 1420 Omani adult subjects.^32^,^33^ The authors analyzed that study using two different definitions of cardiovascular disease (CVD) risk. When CVD risk was identified as the presence of at least two out of three risk factors (hyperglycemia, hypertension and dyslipidemia), the optimal BMI cut-off points for men and women were 23.2 and 26.8, respectively.^32^ Using the Framingham risk score, the optimal cut-off points for men and women were 22.6 and 22.9, respectively.^33^ The use of the first definition resulted in moderate sensitivity (71.0) and poor specificity (53.7) with AUC of 0.65 in men and poor sensitivity (46.8) and moderate specificity (76.5) with AUC of 0.66 in women. Using the Framingham risk score resulted in good sensitivity (80.3), very poor specificity (37.3) and AUC of 0.60 in men, and good sensitivity (84.2), poor specificity (45.1) and AUC of 0.64 in women. Both methods indicated that waist-to-hip ratio (WHR) and waist circumference (WC) were better surrogates to detect CVD risk compared to BMI. One major limitation of that study was the small sample size, which may limit the generalizability of the findings to other Middle Eastern populations. Unfortunately, other diagnostic characteristics of BMI as a tool for obesity diagnosis were not calculated.

Most of the other previous studies that have been conducted in non-Caucasian populations did not assess the misclassification rate.^15^,^24^,^32^–^38^ However, one study conducted in Asian Indians also indicated a high overall misclassification rate, particularly in women.^39^ Those authors concluded that the BMI did not accurately predict overweight in that population.

Several reasons may explain the weakness of BMI as a tool to classify obesity in the Saudi Arabian population. First, BMI does not reflect fatness uniformly in all populations and different ethnic groups.^9^,^39^–^41^ The previous Omani study indicated that WHR and WC better predict CVD risk than BMI.^32^,^33^ This may suggest the importance of including a measure of abdominal obesity in classifying obesity in Middle East populations such as those in Oman and Saudi Arabia. Second, the short stature of Saudi women could be limiting the usefulness of BMI in this population.

Strengths and limitations of this study should be recognized. The very large number of participants provided sufficient cases at each single unit of BMI to assess the significance of association between each BMI unit and the presence of diabetes or hypertension. In contrast, the cross-sectional nature of the survey and the absence of measurements of other relevant obesity-related co-morbidities, such as hypercholesterolemia and hypertriglyceridemia, could be considered as limitations in this study. The sample was a convenience non-random sample. However, it is fairly representative of the target population. When we compared the sub-classification of respondents with the latest census done in the eastern province regarding to age and sex, the characteristics of the study sample were similar.^20^ The relatively low response rate of participants coming for the confirmatory diagnostic test and the reliance on a single screening test using capillary blood glucose may have had an effect on the low performance of BMI in predicting diabetes and hypertension in this study. Also the relatively high values chosen for the definition of diabetes and hypertension for participants who did not come for the confirmatory test may have had a similar negative effect on the performance of BMI. However, >70% of participants who did not come for the confirmatory test for diabetes had IFG or IGT based on CFBS and CRBS. The situation was also similar for hypertension. Whether similar conclusions would be reached had this study been done on a random sample with avoidance of the above-mentioned limitations or not remains to be seen in future studies. Similarly, it is not clear if such conclusion would be obtained in a national sample covering other regions of Saudi Arabia.

In conclusion, the diagnostic usefulness of BMI alone in defining obesity is limited in this large population of Saudi adults in the Eastern Province, for both men and women. Future studies incorporating other measures such as WC, WHR, body fat composition, or a combination of tools, need to be conducted to determine the best method to classify obesity accurately in the Saudi population. It seems likely however that limiting management of obesity to those individuals with a BMI>30 may mean that many Saudis at risk of serious co-morbidities could be missing necessary interventions.
